# Factors associated with last dental visit or not to visit the dentist by Brazilian adolescents: A population-based study

**DOI:** 10.1371/journal.pone.0183310

**Published:** 2017-08-31

**Authors:** Emílio P. da Fonseca, Antonio C. Frias, Fábio L. Mialhe, Antonio C. Pereira, Marcelo de C. Meneghim

**Affiliations:** 1 Department of Community Dentistry, Piracicaba Dental School, FOP/UNICAMP, Graduate Program, University of Campinas, Piracicaba, São Paulo, Brazil; 2 Department of Community Dentistry, Dental School of São Paulo University, FO/USP, São Paulo University, São Paulo, Brazil; 3 Department of Community Dentistry, Health Education and Health Promotion Area of Piracicaba Dental School, FOP/UNICAMP, University of Campinas, Piracicaba, São Paulo, Brazil; 4 Department of Community Dentistry, Preventive Dentistry and Public Health Area of Piracicaba Dental School, FOP/UNICAMP, University of Campinas, Piracicaba, São Paulo, Brazil; University of Washington, UNITED STATES

## Abstract

**Objectives:**

We investigated the factors associated with no dental visit within the last two years by adolescents in the state of São Paulo, Brazil, by using data from the Oral Health Conditions of São Paulo state population Project (SBSP-2015) conducted in 2015.

**Methods:**

This was a cross-sectional epidemiological study with a representative sample of adolescents aged 15 to higher years residing in São Paulo State. The examiners were calibrated and dental visits were measured categorically as 1- Less than 1 year, 2- One to two years, 3 - Three years or more, 4- I have never visited the dentist. Based on the literature we dichotomized the outcome in two groups: response 1 plus 2 against response 3 plus 4. Then, Multilevel Poisson Regression (MPR) was used to estimate the prevalence ratios of last dental visit three years or had never been to a dentist by adolescents compared with those who had visited the dentist within the past two years, with contextual variables as the distal level; sociodemographic variables, mesial; and individual variables, proximal level.

**Results:**

A high percentage of adolescents (84.9%) reported visiting the dentist in the last 2 years. Whereas, 626 (11.6%) had not visited the dentist for over 3 years and 188 (3.4%) had never been. A significantly higher proportion of females than males reported visiting the dentist in the past 2 years (*p* = 0.003). The oral and dental condition was reported as satisfactory by 4,350 respondents (80.6%), and when they accessed the health service, 2,286 (42.3%) went to the public service. Lower mean family income (1.62PR;95%CI;1.36–1.94); ≥ 1,000 inhabitant/Dental Surgeons (1.25PR;95%IC;1.03–1.56);male (1.26PR;95%CI; 1.11–1.43) non-Caucasian ethnicity (Mulatto:1.30PR;95%CI;1.13–1.50 and Black:1.58PR;95%CI;1.29–1.93); dissatisfaction with the oral health condition (1.20PR;95%CI;1.01–1.45),last visit to the public service versus private service (2.26PR; 95%CI;1.91–2.65) and presenting with periodontal disease in the form of dental calculus as the worst situation (1.38PR; 95%CI; 1.16–1.53) were associated with last visit to the dentist.

**Conclusions:**

A high proportion of adolescents had visited the dentist in the last two years. No dental visit within the last two years by adolescents were associated with contextual, health care system, sociodemographic, personal and oral health status, demonstrating that this is a complex phenomenon. Actions to promote regular dental visits by adolescents in Brazil should take these factors into consideration.

## Introduction

Oral health is a basic human right and an important public health issue but is a neglected area of international health [1]. Studies have demonstrated that delaying visit to oral health services increases the risk of poor oral health outcomes [2–5]. Socioeconomic conditions, cost, and a number of individual factors have been identified as potential barriers to accessing dental care [6]. Dental health care services differ greatly among countries in terms of organization, accessibility, availability, and cost. In some countries, full dental health services are readily available through private or public systems [2]. In Brazil, the state offers universal coverage of health services, organized around providing a health care package to all citizens, without suffering financial hardship when paying for them [7]. However, public health care is not one-size-fits-all and this fact is a barrier to the use of public oral health care services worldwide [7].

Studies from many countries, including Brazil, have indicated inequalities in access to oral health and identified factors capable of increasing these inequalities among specific patient groups [8–13]. Factors linked to oral health policies; the structure of services; general and regional socioeconomic and demographic conditions; and collective/individual behavioral contexts have been identified factors influencing delayed visits and non-use of dental services [14–18]. Nonetheless, there is a scarcity of studies investigating the contextual, service and individual factors associated with delaying or no visit the dentist by adolescent populations.

The most recent epidemiological research on oral health conditions in the Brazilian population (SB Brasil-2010) reported that only 16.2% of Brazilian adolescents nationwide and 18.8% in the Southwestern Region had visited a dentist within the past 3 years [19]. Among adolescents, the pattern of dental service use is related to age; sex; socioeconomic condition; beliefs; oral health behaviors, and perception of current oral health condition [9–13]. Understanding the reasons for not visit the dentist by adolescents is essential for strategic planning and efforts to promote good oral health and continuation of regular dental care into adulthood.

Therefore, considering the importance of inequalities in oral health, the aim of this study was to investigate the factors associated with last dental visit three or more years ago by adolescents in the state of São Paulo, Brazil, using data from the Oral Health Conditions Project (SBSP-2015),a survey of the oral health status of the population in the state of São Paulo in 2015.

## Materials and methods

**Fig 1:**
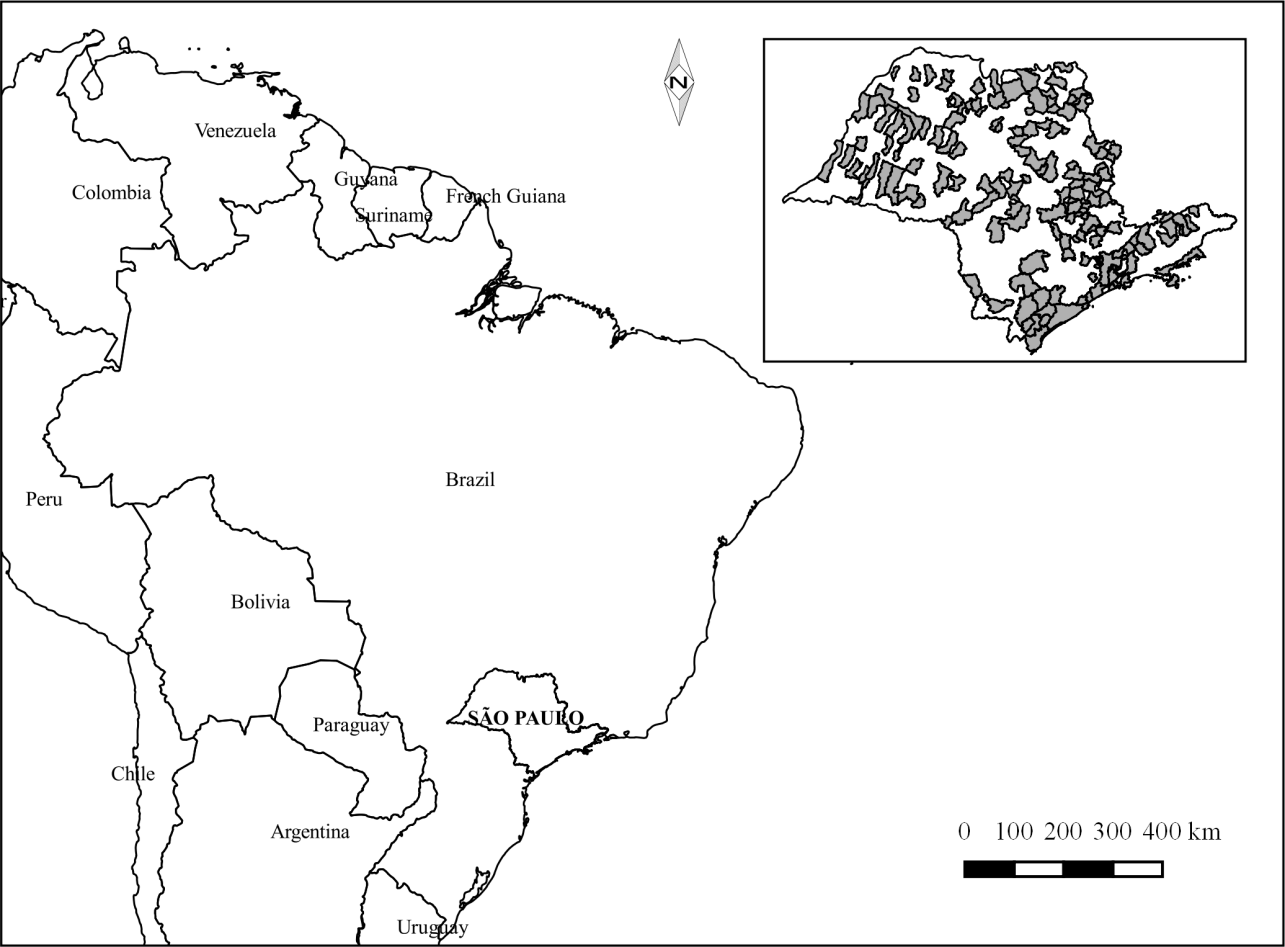
State of São Paulo location and cities selected for the SBSP-2015 survey, Brazil, 2016.

This was a government -sponsored study in the state of São Paulo [20], which had an estimated population of 44,396,484 in 2015, of whom 3,360,982 (7.6%) were in the target age group from 15 to 19 years (Available at http://ibge.gov.br/estadosat/perfil.php?sigla=sp). The age-range from 15 to 19 years represented the group of adolescents in the index age for epidemiological surveys [20] (Fig 1).The authors were responsible for the coordinating the population survey; data collection, and the primary investigators of this study. To ask about the database, all researchers can send an email to apereira@fop.unicamp.br or projetosbsp2015@gmail.com with the following information: name, institution and reason of interest. The data is available at Piracicaba Dental School website http://w2.fop.unicamp.br/sbsp2015/ or Figshare public data repository–Licence CC BY 4.0 with DOI: 10.6084/m9.figshare.5286025.v1.

Subjects were chosen by conglomerate/cluster sampling in two stages, with probabilities proportional to the population size (PPS), taking into consideration the sample weight and effect of design on the respective stages of the draw [20]. The State of São Paulo was stratified into six macro-regions termed “domains,” and in each domain, 33 municipalities were drawn, termed Primary Sampling Units (PSUs), with the exception of Macro 1 (Metropolitan Region of the Capital) where 12 municipalities, in addition to the capital, were drawn. The draws were performed with PPS in each municipality. In the second stage, 390 census sectors (Secondary Sampling Units or SSUs) were drawn, with 2 sectors in each municipality, respecting the proportional probability to the number of inhabitants in the sectors. For the city of São Paulo, 36 sectors were drawn [20]. The sample size was calculated by using the mean values of dental caries; prevalence of periodontal conditions; prevalence of use and need for dental prosthesis; with the respective standard deviations; acceptable error margins (ε); design effects (deff = 2), and non-response rates (NRR = 30%) of the diseases. Finally, a sample of 5,558 adolescents aged 15 to 19 years from State of São Paulo, Brazil, was obtained [20].

Training and calibration processes of the dental teams (dentists and assistants) were conducted by the gold-standard examiner to achieve standardization and agreement among the dental teams. During this phase, examiners studied the codes and criteria, and discussed clinical diagnosis in order to reach an acceptable level of inter-rater agreement statistic Kappa ofover k>0.76 [20].

The dental visits were measured categorically as 1- Less than 1 year, 2- One to two years, 3 - Three years or more, 4- I have never visited the dentist and dichotomized the outcome in two groups: response 1 plus 2 against response 3 plus 4. Then, Multilevel Poisson Regression (MPR) was used to estimate the prevalence ratios of adolescents who confirmed that they had not visited a dentist for over three years or had never been to a dentist compared with those who had visited the dentist within the past two years [18].

Primary Level of MPR included characteristics with Block 1 (Distal Level—contextual variables/municipal variables). The variables of Block 1 were (i) mean family income (available attabnet.datasus.gov.br)divided into 3 categories (> USD 285.00, USD284.00 to 222.00or≤ USD 6221.00),(ii) access to fluoridated water (available at www.cecol.fsp.usp.br) divided into 2 categories according to fluoride content (≥ 0.6 ppm or<0.6 ppm),(iii) ratio of the number of inhabitants in the municipality divided by the number of Dental Surgeons (DS) registered in the municipality (≤ 500 inhabitant/DS,501 to 999 inhabitant/DS or>1000 inhabitant/DS),(iv) municipal Dental Care Index (DCI), [21,22] defined as the ratio of the number of teeth restored to the numerical value of the DMF-T index, and reflecting access to municipal services for dental restoration in relation to tooth decay experience (≥58.0% or<58.0%), and (v) coverage by the Family Health Strategy Team-FHS team (≥ 50.0% or<50.0%). Coverage by a FHS team meant potential access to the primary public oral health care for adolescents [18].

Block 2 (Medial Level-individual variables) contained the variables sex (Female or Male), self-declared skin color according to categorization of the Instituto Brasileiro de Geografia e Estatística (IBGE) Census (Caucasian, Asian, Native Brazilian, Mulatto or Black), and density of persons per room in residence (≤1, 1.1 to 2, or >2.0 persons/room (available at http://cidades.ibge.gov.br).

Block 3 (Proximal Level-individual variables) was composed of individual variables with subjective and objective dimensions, respectively: reports of dissatisfaction with oral and dental conditions (very satisfied, satisfied, or either satisfied or dissatisfied versus dissatisfied or very dissatisfied),payment model for last dental visit (Fee for service, Private health insurance, Others versus Public health insurance), and periodontal disease (no periodontal diseases versus gingivitis, calculus or periodontal pockets). The Community Periodontal Index (CPI), recommended by the World Health Organization (WHO) for research into the prevalence of periodontal problems in adolescents, includes the presence or absence of gingival bleeding, dental calculus and periodontal pockets (shallow and deep) [19,20].

Poisson regression was used to analyze the categorical outcomes, which were expressed as the Prevalence Ratios (PRs) with 95% Confidence Intervals. In the multilevel (hierarchical) model of analysis, the lower medial and proximal levels were adjusted according to the distal level. The variables of the medial level were adjusted to each other and to the distal level; and the variables of the proximal level were adjusted to the distal and medial level and to each other.

**Fig 2:**
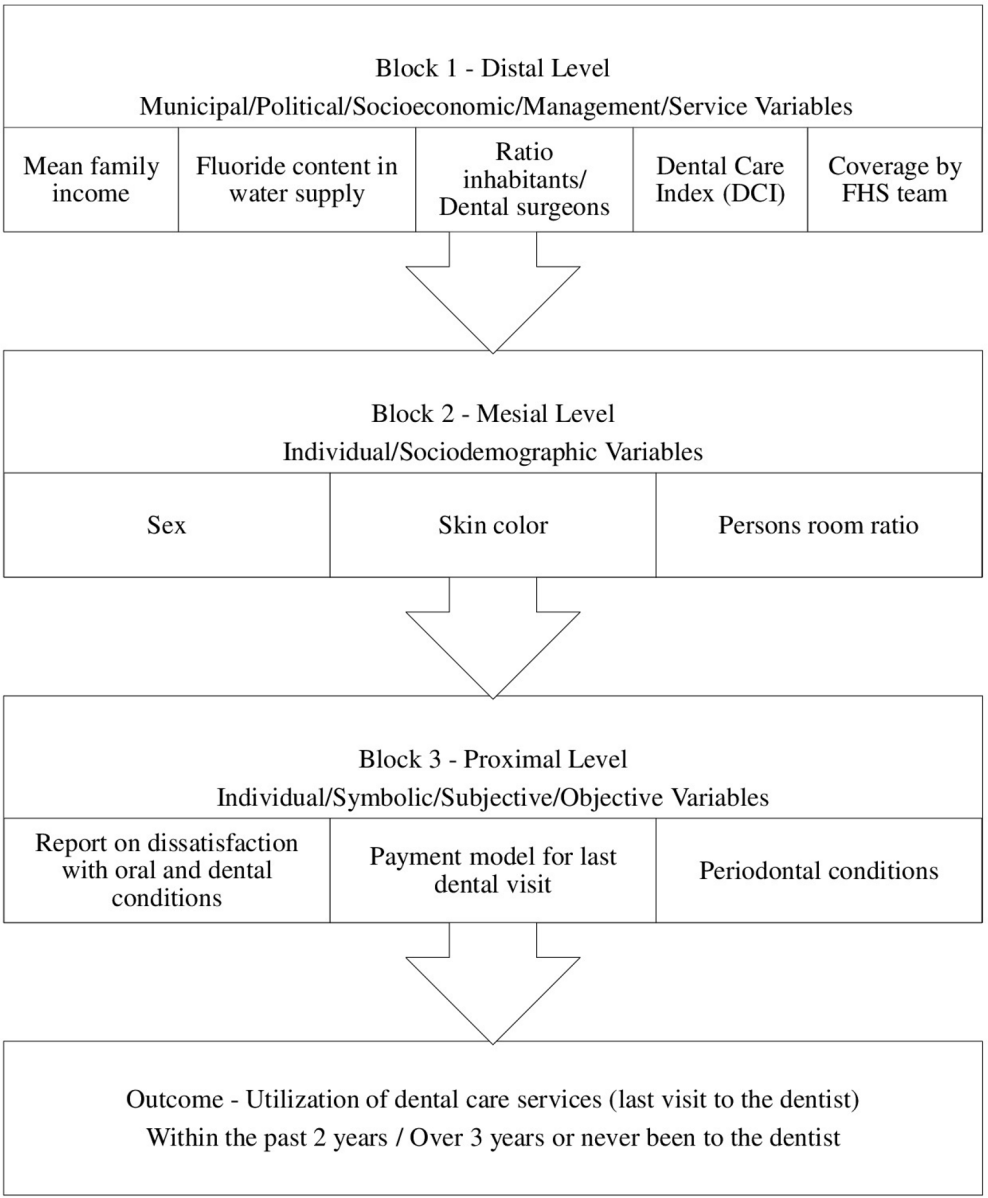
Hierarchical theoretical model for factors associated with use of oral health services by adolescents from 15 to 19 years of age in the State of São Paulo (2016).

The MPR analysis included a model (framework) that incorporated multiple levels of aggregation; corrected standard errors; intervals of confidence, and tests of hypotheses [21, 22]. Once the levels required in the hypothetical model were identified, the authors defined the variables of each level that would be investigated [23–25]. The predictive variables had to represent all the levels contained in the model and the variable criterion had to come from a more proximal level, so that the organization of the variables would follow a hierarchical structure (Fig 2).

Apart from the empty model, two hierarchical models were adjusted with the inclusion of contextual and individual variables. To complete the model, it was assumed that binary responses followed the Poisson distribution; the variables were independent, and that the means and variances were equal [23]. Moreover, in the multilevel models, random effects were considered. The multilevel models were calculated separately for the dependent variable “l visit the dentist within the past two years and last dental visit over three years or never been to the dentist”. The models were first adjusted without inclusion of the dependent variable (empty model) [23]. The quality of adjustment of the models was evaluated by the Log pseudo-likelihood statistics [25]. The Statistical Package for Social Sciences (SPSS version 11.5) and STATA 8.0 were used for all data analyses.

This study was approved by the Research Ethics Committee of the Piracicaba Dental School under Protocol Number 094/2015.

## Results

Of the 5,558 adolescents selected for the study, 5,394 answered the question on the outcome *access*; of these 2,355 (43.7%) were males and 3,039 (56.3%) females. A high percentage of adolescents (84.9%) reported visiting the dentist in the last 2 years. On the other hand, 626 (11.6%) had not visited the dentist for over3 years and 188 (3.4%) had never been to a dentist. The majority of respondents had access to water fluoridated at acceptable levels (4,987, 92.4%).Factors associated with no dental visit within the last two years were lower mean family income; water Fluoridation; coverage by Family Health Strategy (FHS) team. Over 1000 inhabitants / dental surgeons, dental care index, person / room ratio, male, ethnicity (mulatto, black), payment model for last dental visit and dental calculus (Table 1).

**Table 1:** Bivariate analysis for last dental visit according to contextual and individual variables, in adolescents from 15 to 19 years resident in the State of São Paulo, 2015.

Variables	Last visit to the dentist within last 2years (n = 4580)		Last visit to the dentist longer than 2 years or non-use (n = 814)		
	N	%	N	%	p
**Contextual Variables (municipal)**					
Mean family income					
> USD 285.00	1807	87.0	269	13.0	0.075
USD 284.00 to USD 222.00	1763	85.7	294	14.3	
≤ USD 221.00	1010	80.1	251	19.9	0.075
Fluoride content					
< 0.6 ppm	335	82.3	72	17.7	0.916
≥ 0.6ppm	4245	85.1	742	14.9	
Ratio Inhabitants/ DS^a^					
≤ 500 Inhabitants/ DS	1109	83.3	222	16.7	0.186
501 to 1,000 Inhabitants/ DS	2392	85.6	404	14.4	
> 1,000 Inhabitants/ DS	1079	85.2	188	14.8	
Dental Care Index					
< 58.0%	2050	82.8	426	17.2	0.141
≥ 58.0%	2530	86.7	388	13.3	
Coverage by FHS team					
< 50.0%	2435	83.7	473	16.3	0.571
≥ 50.0%	2145	86.3	341	13.7	
**Individual Variables**					
Sex					
Female	2627	86.4	412	13.6	0.003
Male	1953	82.9	402	17.1	
Ethnicity					
Caucasian	2811	87.4	406	12.6	0.001
Asian	46	82.1	10	17.9	
Native Brazilian	10	71.4	4	28.6	
Mulatto	1397	81.9	308	181	
Black	316	78.4	87	21.6	
Person room ratio					
≤1.0	1006	87.4	145	12.6	0.000
1.1 to 2.0	2568	86.1	415	13.9	
>2.0	1007	79.9	254	20.1	
Dissatisfaction with oral and dental conditions					
Very Satisfied / Satisfied / Indifferent	3749	86.2	601	13.8	0.051
Dissatisfied or Very Dissatisfied	741	79.9	186	20.1	
Payment model for last dental visit					
Fee for service / Private health insurance /Others	2668	92.6	212	7.4	0.000
Public health insurance	1882	82.3	404	17.7	
Periodontal conditions					
Healthy	2731	86.9	410	13.1	0.047
Gingivitis	456	83.5	90	16.5	
Calculus	1062	81.1	248	18.9	
Periodontal pocket	331	83.4	66	16.6	

^a^Dental Surgeons.

Over half of the adolescents (2,908, 53.9%) resided in municipalities where coverage by the public primary health care service (FHS) was lower than 50%.A significantly higher proportion of females than males reported visits to the dentist in the past 2 years (*p* = 0.003). Similarly, a significantly higher proportion of respondents with self-reported Caucasian skin color (2,822, 52.1%) had visited the dentist within the past 2 years compared with other ethnicities. The oral and dental condition was reported as satisfactory by 4,350respondents (80.6%), and when they accessed the health service, 2,286 (42.3%) went to the public service. Rates of periodontal diseases were higher in adolescents who had never been to visit the dentist or visit the dentist for longer than 3 years. Of these, 16.5% reported bleeding, 18.95% calculus, and 16.6% presence of periodontal pocket.

In the multilevel model, the following municipal contextual variables were observed to be associated with adolescents not to visit the dentist longer than 3 years or never been to the dentist: residing in municipalities where the mean family income was lower; without access to adequate fluoride content in the local water supply; over 1,000 inhabitants per dental surgeon in the city, a dental care index below that of the State of São Paulo mean (<58%),and living in cities where fewer than half the families were covered by the Family Health Strategy (FHS). At the medial level, last dental visit three or more years ago were more prevalent in young males, adolescents with mulatto or black skin color, and residents of homes with higher density per room (Table 2).

**Table 2:** Bivariate model of unadjusted analysis and hierarchical model adjusted by the distal, medial, and proximal levels associated with last dental visit three or more years ago by adolescents from 15 to 19 years resident in the State of São Paulo, 2016.

	Unadjusted bivariate model^a^		Model adjusted by hierarchical levels^b^	
	Prevalence Ratio IC 95%	P	Prevalence Ratio IC 95%	p
Log pseudo likelihood (empty model)			2353.34	
Mean family income				
≥ USD 285.00	Reference		Reference	
USD284.00 to USD 222.00	1.10 (0.94–1.28)	0.211	1.16 (0.99–1.36)	0.076
≤ USD 221.00	1.53 (1.31–1.79)	0.000	1.62 (1.35–1.94)	0.000
Fluoride content				
≥ 0.6ppm	Reference		Reference	
< 0.6ppm	1.11 (0.92–1.34)	0.254	1.42 (1.13–1.79)	0.003
Ratio Inhabitants/ DS				
≤ 500 Inhabitants/ DS	Reference		Reference	
501 to 1,000Inhabitants/ DS	0.87 (0.74–1.01)	0.061	1.03 (0.88–1.21)	0.682
> 1,000 Inhabitants/ DS	0.89 (0.74–1.06)	0.199	1.27 (1.03–1.56)	0.025
Dental Care Index				
≥ 58.0%	Reference		Reference	
< 58.0%	1.29 (1.14–1.47)	0.000	1.28 (1.12–1.46)	0.000
Coverage by FHS team				
≥ 50.0%	Reference		Reference	
< 50.0%	1.18 (1.04–1.34)	0.009	1.24 (1.09–1.42)	0.001
Log pseudo likelihood (Block 1—Contextual Distal Level)			2328.17	
Sex				
Female	Reference		Reference	
Male	1.26 (1.11–1.43)	0.000	1.26 (1.11–1.43)	0.000
Ethnicity				
Caucasian	Reference		Reference	
Asian	1.42 (0.80–2.50)	0.229	1.49 (0.86–2.57)	0.150
Native Brazilian	2.27 (0.99–5.22)	0.054	2.18 (0.98–4.85)	0.055
Mulatto	1.43 (1.25–1.64)	0.000	1.30 (1.13–1.50)	0.000
Black	1.71 (1.39–2.11)	0.000	1.58 (1.29–1.93)	0.000
Person room ratio				
≤1.0	Reference		Reference	
1.1 to 2.0	1.10 (0.92–1.31)	0.274	1.11 (0.93–1.32)	0.257
>2.0	1.60 (1.32–1.92)	0.000	1.54 (1.27–1.86)	0.000
Log pseudo likelihood (Block 2—Contextual Distal Level + Sociodemographic Medial Level)			2298.52	
Dissatisfaction with oral and dental conditions				
Very Satisfied / Satisfied / Indifferent	Reference		Reference	
Dissatisfied and Very Dissatisfied	1.45 (1.25–1.68)	0.000	1.20 (1.01–1.45)	0.043
Payment model for last dental visit				
Fee for service / Private health insurance /Others	Reference		Reference	
Public health insurance	2.40 (2.05–2.81)	0.000	2.26 (1.91–2.65)	0.000
Periodontal conditions				
Healthy	Reference		Reference	
Gingivitis	1.26 (1.02–1.56)	0.029	1.19 (0.92–1.53)	0.179
Calculus	145 (1.26–1.67)	0.000	1.38 (1.16–1.64)	0.000
Periodontal pocket	1.27 (1.00–1.62)	0.046	1.27 (0.97–1.66)	0.085
Log pseudo likelihood—Block 3 - (Contextual Distal Level + Sociodemographic Medial Level + Proximal Level dimension symbolic/subjective / objective)			2219.86	

^a^Prevalence ratio and 95%confidence interval unadjusted model.

^b^Prevalence ratio and 95% confidence interval (adjusted model, outcome, contextual and individual factors for the hierarchical levels of analysis by Poisson regression).

At the proximal level, the variable dissatisfaction with the oral and dental condition was significantly associated with last dental visit three or more years ago (1.20PR; 95%CI; 1.01–1.45). In addition, last consultation at the public service versus a private service (2.26PR; 95%CI; 1.91–2.65) and presenting with periodontal disease in the form of dental calculus as the worst situation (1.38PR; 95%CI; 1.16–1.53) were associated with no dental visit within the last two years.

In the hierarchical model, there was interaction between the variables of the distal, medial levels compared with last visit to the dentist (proximal level). Moreover, the model adjusted by the hierarchical levels was shown to be more parsimonious when the measurements of quality of adjustment (Log pseudo-likelihood) were compared with the unadjusted model.

## Discussion

This population-based study identified several contextual and individual factors associated with the last visit or never been to the dentist (both in the public and in private sector) by adolescents of State of São Paulo, Brazil.

In 2002, 14.6% of the adolescents residing in Brazil had visited the dentist within the past 3 years, and 25.7% of those who went were motivated by pain [16].Our findings for 2015 in the same state were that there was an increase to 84.9%.

Higher incomes, improved family ties, and more awareness of their family members concern about dental care was associated with a demand for dental services [26–30]. In the present study, the mean family income was associated with use of dental care at some time in the adolescent’s life, corroborating findings of other studies [2,17]. This meant that individuals with better socioeconomic conditions had the resources (health insurance or funds) to pay for a private dental service, with the private sector being responsible for a very significant proportion of oral healthcare coverage [11,12]. In countries with universal health coverage for oral health, including Brazil, it has been observed that access to and use of these services were still affected by socioeconomic inequalities [27,28]. It is important to recognize that the social context is capable of affecting the adolescent population’s quality of life, particularly that of the more vulnerable individuals. Furthermore, association between the variable “ratio of persons per room” and outcome suggested that housing must offer safety, privacy, and personal space to form a cohesive family unit in which oral health problems were deemed priorities. However, studies have shown that residing in poorer areas did not necessarily constitute a barrier to the use of public health services [23].

Adolescents who took longest to see a dentist were from the lowest income level; tended to use public services, and lived in agglomerates. Number of residents may affect family characteristics and prevail over individual characteristics, as well as determine dental services use by adolescents [26]. Differences in the use of oral health services between ethnic groups may reflect the level of social and material deprivation in groups facing economic or social iniquities, particularly non-Caucasian individuals [27].

Oral health service use by adolescents may reflect their level of awareness and expectations as well as their need [27]. These factors may explain the association between negative self-perception of oral health and access to dental services by adolescent resident in the State of São Paulo. Adolescents were also shown to take longer to visit the dentist in municipalities that have ratios higher than 1,000 inhabitants: dental surgeons. Regular visits to the dentist provide opportunities for early diagnosis and treatment of oral diseases, reduce emergency visits, and may result in attitudes towards better prevention and conservation, when compared with patients who only visit dentists when they have a serious problem [27,30].

Surprisingly, the worst stages of periodontal disease (presence of calculus and periodontal pockets) were associated with last dental visit three or more years ago. These stages of disease demand professional intervention and may indicate the lack of access to a dentist. A previous epidemiological survey conducted in the State of São Paulo showed lower ratios of adolescents with gingival bleeding and dental calculus in cities where the level of use of dental services was higher [31]. Poorer individuals, in addition to using dental services less frequently, did not seek them primarily for preventive reasons; this further reduced early detection of disease and the level of less invasive treatment, resulting in worse prognosis [9,31].

A high proportion of adolescents used public dental services, promoted by restructuring of the National Oral Health Policy in 2004 [7]. A previous study reported that the use of dental care at public facilities was more concentrated among the lower socioeconomic groups, while use of private facilities was higher among the better off [28]. Higher educational level was related to a higher level of use of health services, even with restricted family income [16]. Financial limitations may not be the main barrier or reason for not visiting a dentist [8].

Inequalities in oral health persist as a major public health problem [11,26]. Expansion of the public health service network has led to individuals of worse socioeconomic levels having access to these services [18]. In this study, over half of the adolescents lived in municipalities with the low FHS coverage [16] (potential access), which indicated that barriers to accessing primary oral healthcare were still being experienced by adolescents in Brazil. Residing in areas close to a public dental service, in addition to making it easier to visit the dentist, could have a positive impact on the perception of the protective factor of dentistry and favor a higher level of access to primary care [14,18]. However, previous studies have observed no difference in use between individuals who lived in an area covered by the FHS compared with those who did not [14,18].

Universal access to fluoride, especially to fluoridated water, is an important part of public health policies [1]. This access influences oral health in two ways; firstly, levels lower than 0.5 ppm are ineffective for caries prevention, and secondly, adequate levels of fluoride are a protective factor in oral health [24]. In 2009, 99 municipalities (15.3%) in the State of São Paulo almost all with fewer than 50,000 inhabitants did not reach the state average rate of access of 85.1% to fluoridated water [32]. This was probably because small municipalities made fewer investments in improving sanitation, including water fluoridation [32].

The methodological diversity of studies and the number of variables presented in relation to oral healthcare access by adolescents impede direct comparison between previous findings and this study, as there is no methodological standard for studies on this topic. This may be due to the regional diversity in the organization of oral health systems, both in Brazil and elsewhere [31]. Therefore, memory bias cannot be discarded as a confounding variable of the study because this was a cross-sectional study, it was impossible to establish a causal relationship among the studied factors, or to generate qualitative information about past oral healthcare experiences of young users or their parents/guardians. Unhealthy habits such as smoking, eating sweets, and physical inactivity, are associated with fewer visits to dentists and greater curative needs [9], and these factors were not examined in this study. Future research investigating tobacco, alcohol, and drugs as causes for not visit the dentist among adolescents must be conducted.

This study focused on identifying predictive factors for delay three or more years ago or not visit the dentist and thereby provide support for better planning of dental health programs targeting adolescents. The authors were able to identify the complex network of socioeconomic and demographic factors influencing the primary outcome. Concurrent strategies for increasing access to dental services by adolescents including reduction in socioeconomic inequalities (at least with regard to healthcare fees) and expansion of public dental services to guarantee regular visits to the dentist must be adopted. Health planners and professionals in general could encourage prevention of oral diseases in youngsters with greater difficulties accessing dental health services.

## Supporting information

S1 File Survey database of a representative sample of the adolescent population living in the state of São Paulo, Brazil. (DTA)
